# Influence of particle size and fluorination ratio of CF*_x_* precursor compounds on the electrochemical performance of C–FeF_2_ nanocomposites for reversible lithium storage

**DOI:** 10.3762/bjnano.4.80

**Published:** 2013-11-01

**Authors:** Ben Breitung, M Anji Reddy, Venkata Sai Kiran Chakravadhanula, Michael Engel, Christian Kübel, Annie K Powell, Horst Hahn, Maximilian Fichtner

**Affiliations:** 1 Karlsruhe Insititute of Technology (KIT), Institute of Nanotechnology (INT), Hermann-von-Helmholtz-Platz 1, 76344 Eggenstein-Leopoldshafen, Germany; 2 Helmholtz Institute Ulm (HIU) Electrochemical Energy Storage, Albert-Einstein-Allee 11, 89081 Ulm, Germany; 3 Karlsruhe Insititute of Technology (KIT), Karlsruhe Nano Micro Facility (KNMF), Hermann-von-Helmholtz-Platz 1, 76344 Eggenstein-Leopoldshafen, Germany; 4 Karlsruhe Institute of Technology (KIT), Institute for Inorganic Chemistry, Engesserstrasse 15, D-76128 Karlsruhe, Germany

**Keywords:** conducting carbon, conversion material, enregy-related, graphite fluoride, lithium battery, iron fluoride

## Abstract

Systematical studies of the electrochemical performance of CF*_x_*-derived carbon–FeF_2_ nanocomposites for reversible lithium storage are presented. The conversion cathode materials were synthesized by a simple one-pot synthesis, which enables a reactive intercalation of nanoscale Fe particles in a CF*_x_* matrix, and the reaction of these components to an electrically conductive C–FeF_2_ compound. The pretreatment and the structure of the utilized CF*_x_* precursors play a crucial role in the synthesis and influence the electrochemical behavior of the conversion cathode material. The particle size of the CF*_x_* precursor particles was varied by ball milling as well as by choosing different C/F ratios. The investigations led to optimized C–FeF_2_ conversion cathode materials that showed specific capacities of 436 mAh/g at 40 °C after 25 cycles. The composites were characterized by Raman spectroscopy, X-Ray diffraction measurements, electron energy loss spectroscopy and TEM measurements. The electrochemical performances of the materials were tested by galvanostatic measurements.

## Introduction

Lithium-ion batteries are key energy storage systems for portable and mobile electric devices. However, for applications that need high energy densities, current insertion-based lithium-ion batteries do not match the targets for such systems [1–4]. As a perspective, energy storage materials that are based on conversion reactions may offer high theoretical capacities and high theoretical energy densities for hydrogen storage and for electrochemical storage in batteries [5]. Compared to state-of-the-art insertion cathode materials with specific capacities of 150 mAh/g for LiCoO_2_ [6] up to 170 mAh/g for LiFePO_4_ [7] conversion cathode materials can theoretically provide more than three times higher theoretical specific capacities. The theoretical capacity of the herein investigated FeF_2_/Li^+^ conversion system amounts to 571 mAh/g [8]. This mainly results from a utilization of several oxidation states of the active metal that allows for a multi-electron process per redox step compared to only one-electron processes in the insertion materials [9–11].

An early example for conversion reactions in batteries was demonstrated by Poizot et al. who used transition-metal oxides as anode materials [9]. Metal fluorides are also prominent examples as they reversibly react with lithium at relatively high voltages so that they can be used as cathode materials [5,8,12–16]. Fluorine is the lightest and smallest halogen in the periodic table of elements, which is a precondition to achieve a high gravimetric energy density in batteries. Iron fluorides are attractive as electrode materials because of their large abundance, low cost and low toxicity. However, because of the electrically insulating nature of metal fluorides, a well conducting nanoscale matrix is required to ensure the electron transport to the active material. Micrometer-sized metal fluoride particles are too big to accommodate the transfer of electric charge, and their capacity fades rapidly with cycling. Hence, a nanoscale dispersion of the material in the matrix is a precondition for its electrochemical activity [17–18]. In addition, volume changes that result from phase conversion of the active material during charging and discharging may lead to cracks in the particles and result in poor cyclic stabilities. For this reason, mostly carbon materials have been used as conducting matrix as well as to buffer the volume changes.

Various methods have been described in the literature to synthesize carbon–metal fluoride nanocomposites. For example, carbon–iron fluoride nanocomposites, which show superior electrochemical performance during the initial cycling, have been synthesized by high energy ball milling graphite and iron fluoride [17–19]. However, the major drawback of current conversion materials systems is their low cyclic stability during an extended number of cycles. Considerable efforts have been made to understand and optimize the electrochemical performance of the metal fluoride conversion systems [20–33].

Recently, conversion systems with excellent cyclic stabilities were synthesized through the pyrolysis of metallocenes with LiF, in which agglomerates of LiF and transition metal nanoparticles encapsulated in layers of graphitic carbon were formed. The agglomerates are interlinked by multiwall carbon nanotubes which are formed in situ [34–36]. Although these systems enhanced the cycling stability of the conversion reaction greatly because of the tight embedding of iron nanoparticles in the carbon matrix, their specific capacity was about 250 mAh/g, which is only one third of the theoretical value.

To improve the capacity and to still benefit from a stabilizing and tightly attached carbon matrix, a new solid-state chemical synthesis, which is based on a reaction between CF*_x_* and Fe(CO)_5_ to produce graphitic carbon–FeF_2_ nanocomposites at 250 °C, was developed recently [37]. Fe(CO)_5_ evaporates at 103 °C [38] and decomposes at temperatures above 120 °C [39]. In this way atomic sized Fe(0) nuclei are generated. These Fe(0) particles obviously react inside the CF*_x_* matrix and produce FeF_2_ nanoparticles by reducing the CF*_x_* carbon backbone to graphitic carbon in a reactive intercalation process. The final material contains crystallites of FeF_2_ with diameters of a few nanometers, which are closely packed and embedded between graphitic carbon sheets. The graphitic carbon enwraps the formed FeF_2_ nanocrystallites and provides an electrical contact between the insulating FeF_2_ particles and the collector. The overall reaction follows Equation 1:

[1]
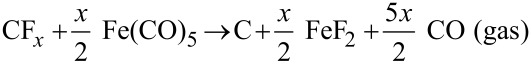


It was also shown that ball milling of CF*_x_* as pretreatment significantly influences the electrochemical performance of the C–FeF_2_ nanocomposites. The electrochemical properties of these nanocomposites likely depend on the amount and type of carbon present in the nanocomposites. In our previous studies we used only graphite fluoride with a fluorine to carbon ratio of 1.1. This resulted from the reaction with Fe(CO)_5_ in 20 wt % of carbon related to the total mass of the composite. In the present work we focus on an optimization of properties by varying the CF*_x_* pretreatment and by varying the amount of carbon in the nanocomposites using different precursors with a variable C to F ratio.

## Experimental

Ball milling of the CF*_x_* precursor was performed in a sealed tungsten carbide vial under inert conditions. The CF*_x_* powder was ball-milled for 2 h, with a ball to powder ratio of 24:1 and milling speeds of 200, 300, and 400 rpm.

To adjust the active material to carbon ratio in the products, different graphite fluoride samples were used with different fluorine to carbon ratios, which correspond to *x* in the reaction equation. The synthesis of the C–FeF_2_ nanocomposites was performed in a tubular stainless steel reactor with metal fittings (VCR®). In a typical synthesis, 0.25 g graphite fluoride (CF_0.5_, CF_0.7_, CF_1.0_, Alfa Aesar, 99%; CF_1.1_, Sigma Aldrich, 99.9%) and the required amount of Fe(CO)_5_ (Sigma Aldrich, 99%) were filled in the reaction vessel inside an argon-filled glove box. The amount of iron pentacarbonyl used for the synthesis was calculated for a complete reaction with the inserted CF*_x_* to FeF_2_. The vessel was closed and heated to 250 °C at a heating rate of 5 K/min and kept at this temperature for 24 h in a horizontal tube furnace. Afterwards, the reactor was let to cool down to room temperature. The pressure was released carefully and the remaining powder was collected under argon atmosphere. The black powder was used without further purification.

Transmission electron microscopy analysis was carried out on an image corrected Titan 80-300 (FEI) operated at 80 kV and equipped with a Tridiem 963 imaging filter (Gatan) for EEL spectroscopy with a nominal energy resolution of 0.8 eV. For the TEM analysis, the dry powders were distributed on holey carbon coated copper grids (Quantifoil).

Powder X-ray diffraction (XRD) patterns were recorded in a 2θ range of 10–40° by using a Philips X’pert diffractometer with Mo Kα radiation. Raman spectroscopy was performed with a confocal Raman microscope (CRM200, WITec). As excitation light source a HeNe gas laser from JDS Uniphase was used at a wavelength of 632.8 nm. The beam was focused through a 100× objective onto the sample. The Raman-scattered light was separated from the laser excitation light by using a holographic notch filter, and spectrally analyzed by using a grating spectrograph and a Peltier-cooled charge coupled device.

Electrochemical studies were performed in Swagelok® type cells. Each cathode material was tested 2–3 times, also at different temperatures and at different current densities. The variation of the obtained specific discharge capacities was always less than 30 mAh/g below or above the presented values for cathode materials cycled under the same conditions. The electrode fabrication and the building of electrochemical cells were done in an argon-filled glove box. The electrodes were fabricated by mixing the synthesized material and polyvinylidene fluoride (PVDF) in the mass ratio 90:10. A slurry containing the above mixture was prepared by using *N*-methyl-2-pyrrolidinone. It was spread on a stainless steel (SS) foil (area: 1.13 cm^2^) and dried on hot plate at 160 °C for 12 h. Typically, each electrode contained 3–4 mg of active material. Lithium foil (Goodfellow, 99.9 %) was used as the negative electrode, and a borosilicate glass fiber sheet was used as separator. The sheet was saturated with 1 M LiPF_6_ in 1:1 ethylene carbonate (EC)/dimethyl carbonate (DMC) (LP30, Merck), which was used as electrolyte. The cells were placed in an incubator (Binder) to maintain a constant temperature of 25 ± 0.1 °C or 40 ± 0.1 °C. The electrochemical studies were carried out using an Arbin battery cycling unit.

## Results and discussion

### Optimization of ball milling conditions

It was shown that a pretreatment of the CF*_x_* precursor directly influences the electrochemical performance of the resulting products [37]. When ball-milled CF*_x_* was used for the reaction, a significant enhancement of the capacity of the cathode material was observed. To compare different ball-milled products of CF_1.1_, the ball milling time of the graphite fluoride was set to 2 h for each sample, at rotation speeds which were 200 rpm, 300 rpm and 400 rpm. After the reaction with iron pentacarbonyl, these samples gave four different cathode materials hereafter named as C(FeF_2_)_0.55_, C(FeF_2_)_0.55__200, C(FeF_2_)_0.55__300, C(FeF_2_)_0.55__400 for unmilled CF_1.1_, and CF_1.1_ milled at 200, 300, and 400 rpm, respectively.

**Figure 1:**
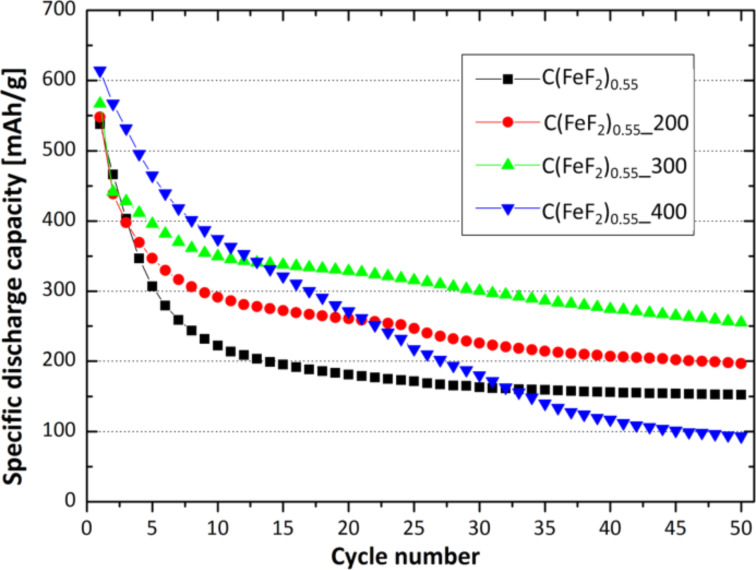
Cycling behavior of C(FeF_2_)_0.55_, C(FeF_2_)_0.55__200, C(FeF_2_)_0.55__300 and C(FeF_2_)_0.55__400. The materials were cycled with a current density of 23 mA/g between 1.3 V and 4.3 V.

Figure 1 shows the cyclic capacity of the C(FeF_2_)_0.55_, C(FeF_2_)_0.55__200, C(FeF_2_)_0.55__300 and C(FeF_2_)_0.55__400 samples. The materials were cycled at a current density of 23 mA/g between 1.3 V and 4.3 V. The data reveals a big influence of the ball milling conditions of CF_1.1_ on the cycling behavior of the nanocomposites. The samples with the CF_1.1_ precursor ball-milled at 300 rpm showed the highest capacities upon cycling. The first discharge capacity increased with increasing ball milling speed of the used CF_1.1_ precursor. The irreversible capacity loss (ICL) during cycling refers to the amount of capacity which cannot be retained in the following cycle. That means, a low or decreasing ICL is the precondition for a stable cycling of the material. For C(FeF_2_)_0.55__400 the ICL did not decrease during cycling, which leads to a decreasing cycling stability for this material, even if the first ICL only amounts to 47 mAh/g which is the lowest ICL for all investigated materials. For C(FeF_2_)_0.55_, C(FeF_2_)_0.55__200 and C(FeF_2_)_0.55__300 the capacity faded much more slowly after the first few cycles, and in the case of C(FeF_2_)_0.55__300 the capacity after 50 cycles (255 mAh/g) reached the highest value compared to the other materials.

### Variation of carbon content

In order to investigate the influence of the carbon content on the electrochemical performance of the nanocomposite, different graphite fluorides (CF_0.5_, CF_0.7_, CF_1.0_ and CF_1.1_) were used as precursors. The materials were ball-milled at 300 rpm for 2 h as this was the best milling condition we could find with respect to the electrochemical performance. Other milling conditions were tested for all materials, but the obtained products showed the best cycling stability and specific capacity when ball-milled with 300 rpm. The ball-milled precursor was used to react with a stoichiometric amount of Fe(CO)_5_ at 250 °C for 24 h. The resulting products were named as C(FeF_2_)_0.25__300, C(FeF_2_)_0.35__300, C(FeF_2_)_0.5__300 and C(FeF_2_)_0.55__300, for CF_0.5_, CF_0.7_, CF_1.0_ and CF_1.1_ respectively. The calculated quantity of active material and carbon in each nanocomposite is presented in Table 1.

**Table 1:** Graphite fluoride precursors and composition of the respective products.

used graphite fluoride precursor	FeF_2_ wt % in product	C wt % in product	designation of the related product

CF_0.5__300	61	39	C(FeF_2_)_0.25__300
CF_0.7__300	73	27	C(FeF_2_)_0.35__300
CF_1.0__300	80	20	C(FeF_2_)_0.5__300
CF_1.1__300	81	19	C(FeF_2_)_0.55__300

**Figure 2:**
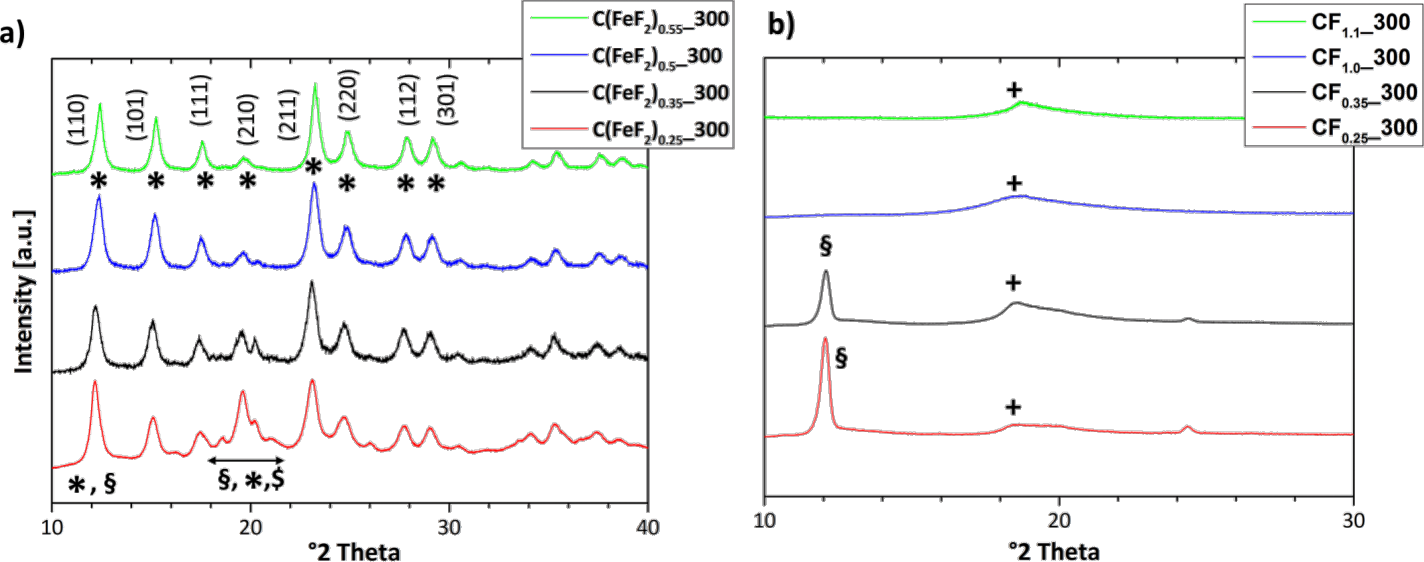
XRD pattern (Mo Kα) of: a) Nanocomposites with different C/F ratio, b) CF*_x_* precursors. *:FeF_2_; §:C (graphite); +:**CF*_x_***; $:iron carbide.

The X-ray diffraction patterns of the nanocomposites are shown in Figure 2a. All nanocomposites show diffraction peaks that correspond to the FeF_2_ rutile structure. However, differences between the patterns can be noticed in the region around 20°. The XRD pattern of C(FeF_2_)_0.25__300 shows an increased intensity of the (210) peak and some additional peaks with lower intensities between 19° and 21°, which result from a graphitic type of carbon. The increase in intensity of the FeF_2_(210) peak is the result of an overlapping FeF_2_(210) signal, a graphite signal and different iron carbide signals. In the XRD patterns of nanocomposites synthesized from higher fluorinated CF*_x_*, the graphite signal and the iron carbide signals decrease, which correspondingly leads to a decreased intensity at the FeF_2_(210) peak. A change of the intensity ratio of the first two FeF_2_ peaks ((110)/(101)) can be noticed as well. It is decreasing for materials with a higher content *x* of fluorine. Due to the overlap of the FeF_2_(110) and the graphite peak, the signal at 12.2° has a higher intensity for composites with increasingly crystalline graphitic domains, which leads to a higher ratio between the first two peaks. Hence, the ordered graphitic domains in the nanocomposites seem to decrease for higher *x* in the used CF*_x_* precursors.

CF*_x_* precursors with different *x* show different structures and types of bonding. Lower fluorinated graphite fluorides (lower *x*) lead to compounds, which contain carbon that is more graphitic in nature [40]. This tendency can also be seen in Figure 2b, in which XRD patterns (Mo Kα) are shown of the different types of CF*_x_*. The data indicate that the structure of the CF*_x_* precursors directly influences the nature of carbon in the synthesized nanocomposites.

**Figure 3:**
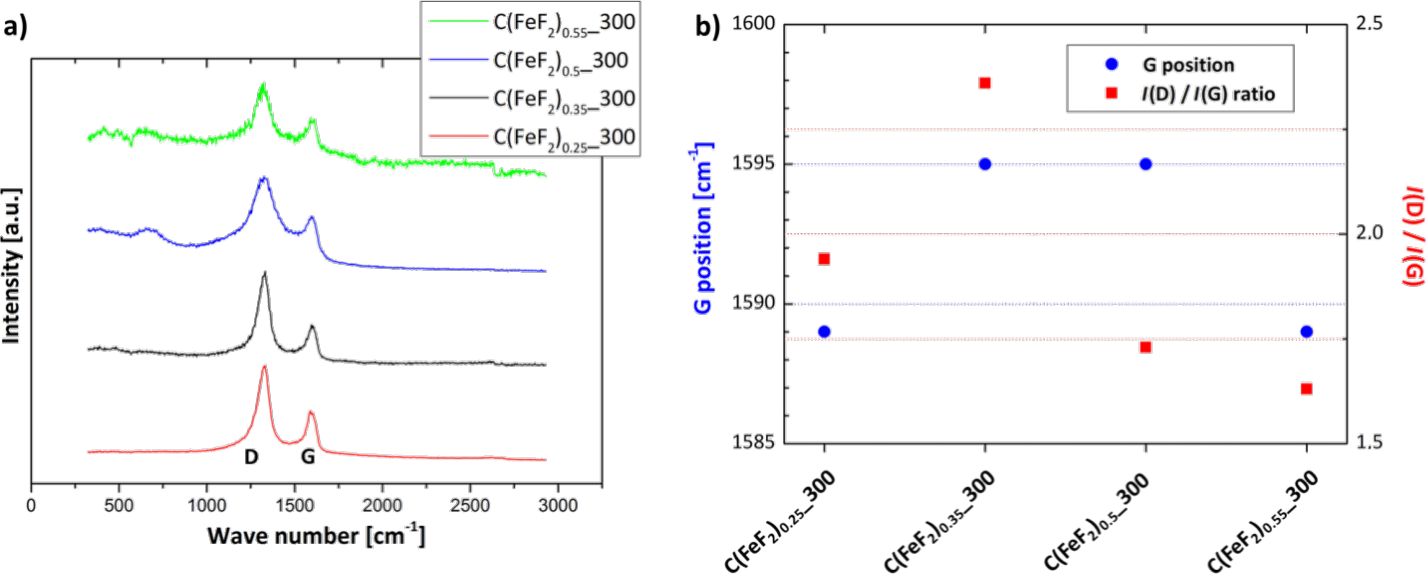
Measured Raman spectra and G-mode shifts of the different nanocomposites.

The variation of the carbon structure in the nanocomposites was further investigated by Raman spectroscopy. Figure 3a shows the Raman spectra of the different nanocomposites, and Figure 3b shows the results of an analysis of the spectra. The position and the ratio of the D and G mode (*I*(D)/*I*(G)) in a Raman spectrum of carbon characterizes the structure and the order of the investigated carbon [41]. Ferrari et al. reported a model to characterize and classify different carbon structures [42–43]. According to this model two different types of carbon are present. Graphite shows a G-mode position of about 1580 cm^−1^ and a *I*(D)/*I*(G) ratio of 0.25. Nanocrystalline graphite exhibits a G-mode position of about 1600 cm^−1^ and an increased *I*(D)/*I*(G) ratio. For C(FeF_2_)_0.25__300, a G-mode position of 1589 cm^−1^ and a *I*(D)/*I*(G) ratio of 1.94 can be noticed. Thus, the nature of carbon in C(FeF_2_)_0.25__300 does not fully match with the bulk graphite characteristics. The properties are shifted towards those of nanocrystalline graphite. With a G-mode position of 1595 cm^−1^ and a *I*(D)/*I*(G) ratio of 2.36 the spectra of C(FeF_2_)_0.35__300 matches with the description of nanocrystalline graphite. For C(FeF_2_)_0.5__300 the same G-mode position was measured, but the *I*(D)/*I*(G) ratio decreased to 1.73. During a transition from nanocrystalline graphite to amorphous carbon the VDOS (vibrational density of states) of graphite changes, the D-mode intensity decreases and the G mode retains its intensity, which results in a decreased *I*(D)/*I*(G) ratio [42]. This tendency is continued with a further decrease of the *I*(D)/*I*(G) ratio (1.63) for C(FeF_2_)_0.55__300 which, in addition, shows a downshift of the G position to 1589 cm^−1^.

**Figure 4:**
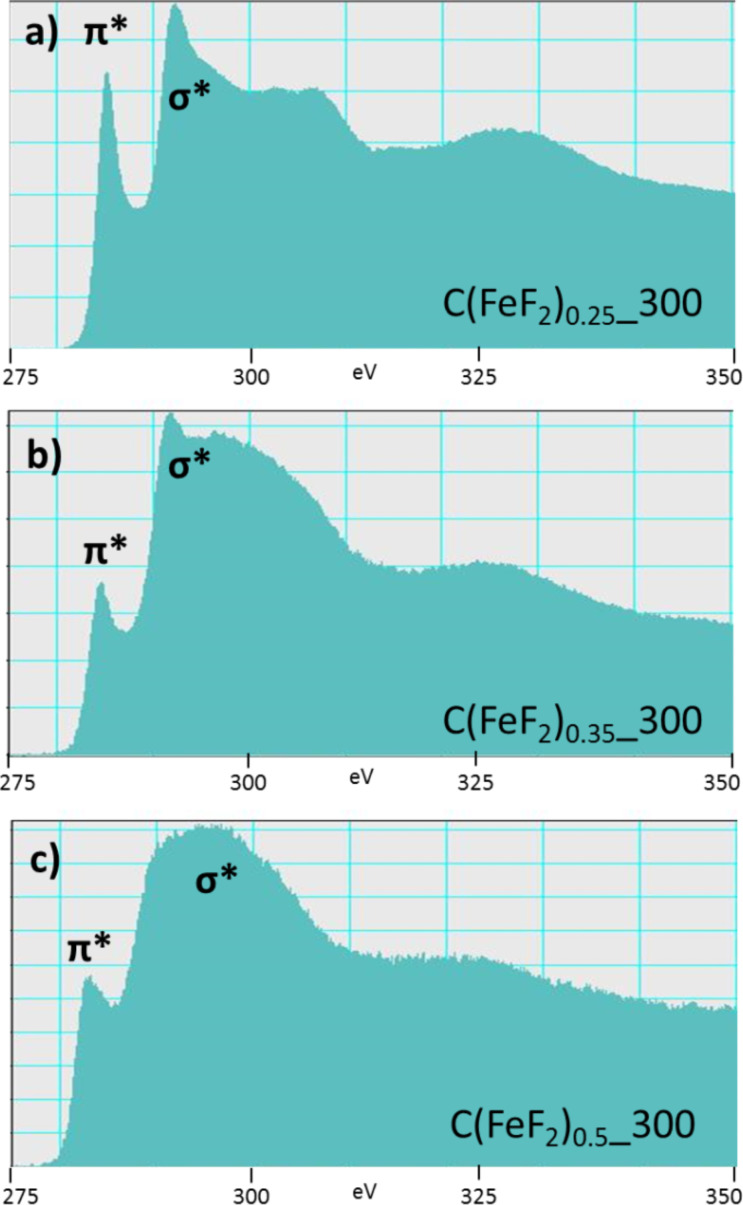
C K-edge EEL spectra of compounds with different carbon contents.

In addition, EEL spectroscopy was performed to further elucidate on the carbon structure. The EEL spectra confirmed the data previously obtained with Raman spectroscopy about the characteristics of the carbon structure. The loss of the distinct sharp structure in the energy-loss near edge structure (ELNES) of the C K-edge (Figure 4) signifies a reduced order of the graphitic carbon matrix [44–46]. At the same time, the peaks, resulting from the transition of the electrons from the π to the π* or σ* band, increase for the products prepared with precursors with a lower C/F ratio. These peaks indicate the presence of a conjugated π system. That means, the choice of the CF*_x_* precursor before the reaction with Fe(CO)_5_ will determine the graphitic character of the carbon matrix.

**Figure 5:**
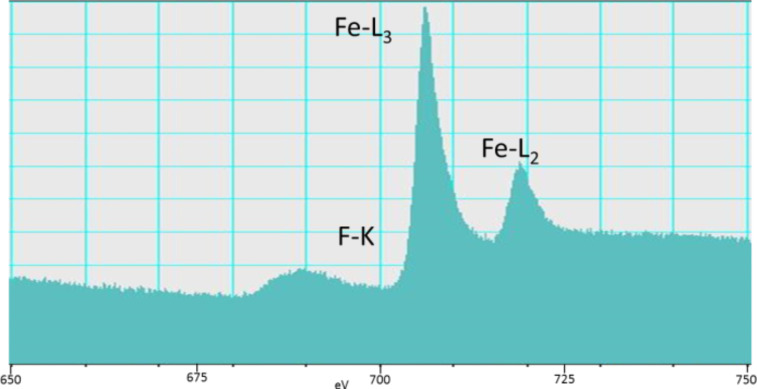
EEL spectra of C(FeF_2_)_0.25__300. The spectrum shows the F K-edge and the Fe L3- and Fe L2-edges.

The EEL spectra from the F K-edge, Fe L3-edge and Fe L2-edge (Figure 5) showed no difference between the various samples prepared with different CF*_x_* precursors and are in good agreement with FeF_2_ [47–48]. (See Supporting Information File 1 for L3/L2 intensity ratio data)

TEM and SAED measurements were made to investigate the microstructure and morphology of the nanocomposites.

**Figure 6:**
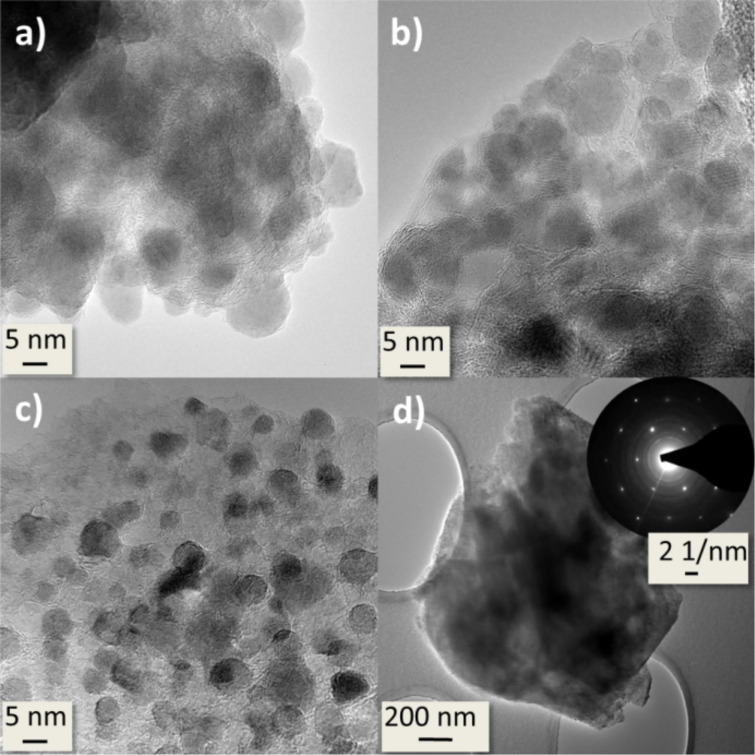
TEM and SAED pictures of a) C(FeF_2_)_0.5__300, b) C(FeF_2_)_0.35__300, c) C(FeF_2_)_0.25__300 and d) one complete particle with SAED pattern of C(FeF_2_)_0.25__300.

Figure 6 shows TEM and SAED pictures of the nanocomposites. The material consists of graphitic carbon with embedded FeF_2_ nanoparticles. Figure 6 a–c show images of the composites C(FeF_2_)_0.5__300, C(FeF_2_)_0.35__300 and C(FeF_2_)_0.25__300, respectively. In comparison, in the C(FeF_2_)_0.5__300 system, the FeF_2_ particles are packed most densely. The FeF_2_ particles in the C(FeF_2_)_0.25__300 system have smaller diameters, mostly below 5 nm, and are more dispersed by the graphitic layers, which can be because of the higher atomic percentage of carbon. While the FeF_2_ particle size increases slightly from C(FeF_2_)_0.25__300 over C(FeF_2_)_0.35__300 to C(FeF_2_)_0.5__300 (below 5 nm at C(FeF_2_)_0.25__300 to around 9 nm at C(FeF_2_)_0.5__300) no visible size-changing effects between C(FeF_2_)_0.5__300 and C(FeF_2_)_0.55__300 could be found. Despite the absence of a change in the particle sizes the electrochemical behavior during cycling is very different between those samples. Therefore we attribute the different electrochemical behavior in all samples to the structural change of the carbon matrix and not to an effect which solely comes from the different FeF_2_ particle size. The graphitic nature of the carbon was evident also in the SAED data. As can be seen in Figure 6d, a highly ordered crystalline structure of graphitic carbon, clearly indicated by the hexagonally arranged spots in the SAED, is shown, when the SAED pattern was taken from the particle surface. The diffraction rings in the picture can be assigned to the FeF_2_ rutile structure.

**Figure 7:**
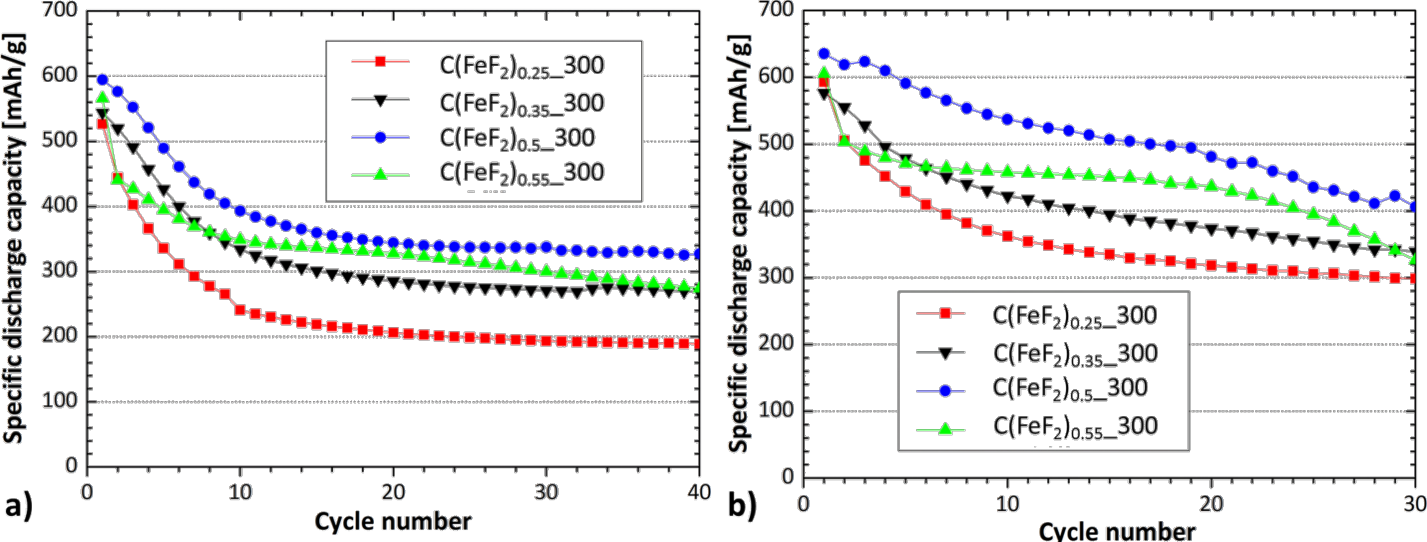
Discharge capacities at: a) 25 °C, b) 40 °C. The samples were cycled with a current density of 25 mA/g.

**Figure 8:**
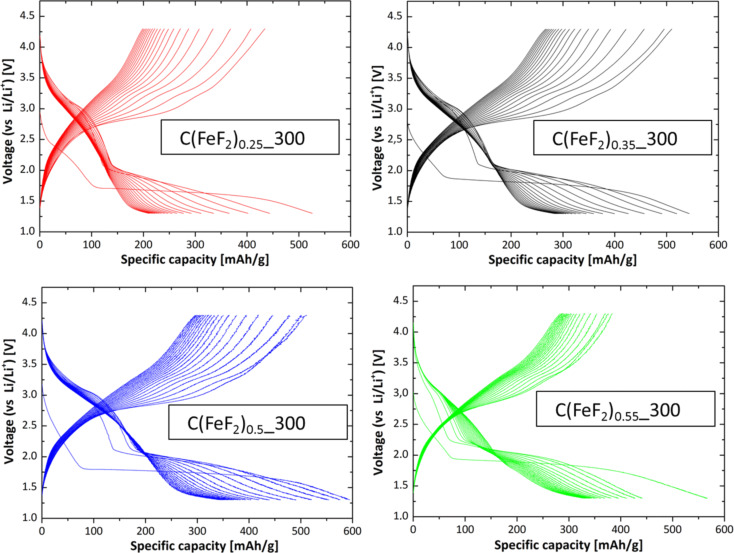
Charge/discharge profiles for the first 20 cycles of the nanocomposites at 25 °C. The samples were cycled with a current density of 25 mA/g.

In galvanostatic measurements, the nanocomposites were cycled at different temperatures with a current density of 25 mA/g between 1.3 V and 4.3 V (Figure 7). C(FeF_2_)_0.5__300 showed the highest capacity and lowest ICL after a few cycles, which led to a high stability of the capacity for the first 40/30 cycles at 25/40 °C. At 40 °C no convergence to a stable capacity value was observed, instead the capacity faded almost linearly. The first discharge capacities also reached their maximum with C(FeF_2_)_0.5__300 as cathode material, and faded for higher or lower contents of active material. The first discharge capacity of C(FeF_2_)_0.5__300 at 40 °C reached a value of 635 mAh/g, which is beyond the theoretical value of an FeF_2_/Li conversion system (571 mAh/g). This overcapacity is the consequence of an electrochemical reaction between unreacted CF_1.0__300 and Li^+^. Graphite fluoride is known to react with lithium to carbon and lithium fluoride between 2.0 V and 3.0 V [49]. This reaction can be seen in the discharge profile of the material (Figure 8). If the capacity that we attribute to the reaction of graphite fluoride with lithium is subtracted from the first discharge capacity of 635 mAh/g, a capacity value is obtained that almost coincides with the theoretical value of the synthesized FeF_2_. The discharge capacity which can be related to the reaction of CF*_x_*, is indicated by a slope at the beginning of the first discharge cycle at the discharge profiles (Figure 8).

**Figure 9:**
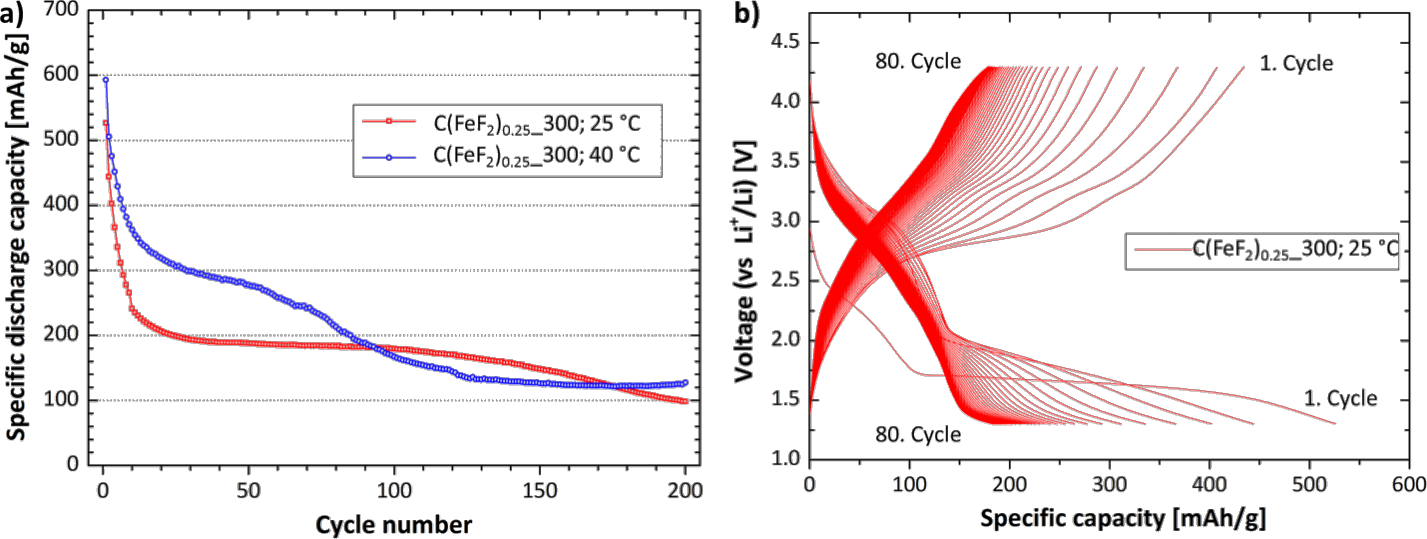
Long time cycling of C(FeF_2_)_0.25__300. a) Specific discharge capacity at different temperatures, b) Charge/discharge profile. The samples were cycled with a current density of 25 mA/g.

Cells with C(FeF_2_)_0.5__300 as cathode material showed the highest capacity, but were lacking in cyclic stability. This capacity behavior was observed for the nanocomposites, which contain a more amorphous type of carbon (C(FeF_2_)_0.5__300, C(FeF_2_)_0.55__300). Contrary to that, the cyclic stability increased for nanocomposites with a higher graphitic carbon content and for lower temperatures (C(FeF_2_)_0.25__300, C(FeF_2_)_0.35__300). Figure 7 clearly shows that, in general, a higher working temperature increased the capacity but affected the cyclic stability of the test cells. Cells built with C(FeF_2_)_0.25__300 as cathode material proved to be the most stable systems for long time measurements. Figure 9 shows the cells cycled at 25 °C and 40 °C for 200 cycles. The residence time of the electrode material in such a cell was around 80 days.

## Conclusion

In conclusion, studies regarding the pretreatment and the C/F ratio of the CF*_x_* precursors for carbon–FeF_2_ nanocomposites for reversible lithium storage as well as with respect to the electrochemical performance and the carbon structure of these nanocomposites were performed. The main reaction and processes during the first and the subsequent cycles were elucidated.

We have optimized the pretreatment and the C/F ratio of the CF*_x_* precursor. Galvanostatic tests of nanocomposites with a more amorphous type of carbon matrix (CF_1.1_; 300 rpm ball-milling speed; 40 °C) showed a capacity of 436 mAh/g after 25 cycles while the nanocomposites with a more graphitic matrix (CF_0.5_; 300 rpm ball-milling speed; 25 °C) showed a stable capacity between 150 mAh/g and 200 mAh/g for more than 150 cycles. The structure of the conducting carbon matrix seems to have a great influence on the electrochemical behavior. Raman measurements showed a transition from graphitic carbon, over nanocrystalline graphite to a more amorphous type of graphitic carbon for the nanocomposites synthesized with different compositions CF*_x_.* A higher graphitic character of the carbon matrix was found for materials produced with CF*_x_* precursors with of a lower F/C ratio). These results were confirmed by EELS and SAED measurements.

## Supporting Information

File 1 Detailed experimental data.
