# Supplementary material for: Influence of particle size and fluorination ratio of CFx precursor compounds on the electrochemical performance of C–FeF2 nanocomposites for reversible lithium storage
Source: Beilstein J Nanotechnol. 2013 Nov 1;4:705–13. doi: 10.3762/bjnano.4.80 (PMC3869371; doi:10.3762/bjnano.4.80)
Supplement: File 1 — Detailed experimental data. [file Beilstein_J_Nanotechnol-04-705-s001.pdf]

Supporting Information

for

Influence of particle size and fluorination ratio of CF_x precursor compounds on the electrochemical performance of C–FeF_2 nanocomposites for reversible lithium storage

Ben Breitung*[1], M. Anji Reddy[1], Venkata Sai Kiran Chakravadhanula[1,2], Michael Engel[1], Christian Kübel[1,2,3], Annie K. Powell[1,4], Horst Hahn[1,2] and Maximilian Fichtner*[1,2]

Address: [1]Karlsruhe Insititute of Technology (KIT), Institute of Nanotechnology (INT), Hermann-von-Helmholtz-Platz 1, 76344 Eggenstein-Leopoldshafen, Germany; [2]Helmholtz Institute Ulm (HIU) Electrochemical Energy Storage, Albert-Einstein-Allee 11, 89081 Ulm, Germany; [3]Karlsruhe Insititute of Technology (KIT), Karlsruhe Nano Micro Facility (KNMF), Hermann-von-Helmholtz-Platz 1, 76344 Eggenstein-Leopoldshafen, Germany and [4]Karlsruhe Institute of Technology (KIT), Institute for Inorganic Chemistry, Engesserstrasse 15, D-76128 Karlsruhe, Germany

E-Mail: Ben Breitung* - ben.breitung@kit.edu;

Maximilian Fichtner* - maximilian.fichtner@kit.edu

*Corresponding author

Detailed experimental data

The L3/L2 intensity ratios of the different composites have been studied and showed decreasing ratios with increasing carbon content. These ratios lie in the range of the values of where FeF_2, as predicted in the work of Cosandey et al. [1]. Further, an increasing carbon content leads to an increasing amount of iron carbides with iron in low valence compounds, which leads to a lower L3/L2 ratio. The XRD data, which do not show any hints of the rhombohedral crystal structure of iron(III) fluoride, is in a good agreement with the TEM data that show the rutile structure of FeF_2.

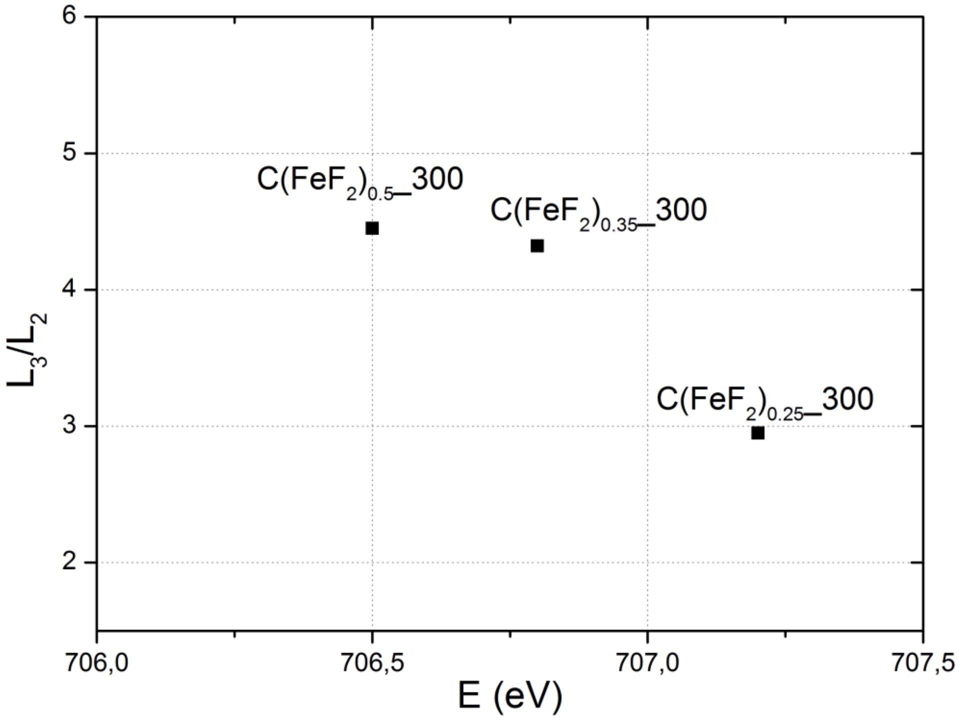

Figure S1: L3 peak energy positions and L3/L2 intensity ratios of the different compounds.

Table S1: Experimental data displayed in Figure S1.

$C(FeF_2)_{0.25}$_300					
	Area	FWHM	Height	Ratio	Peak Position
Fe(L3)	4.079	3.632	0.429	2.95	707.2
Fe(L2)	1.560	4.432	0.145		719.7

$C(FeF_2)_{0.35}$_300					
	Area	FWHM	Height	Ratio	Peak Position
Fe(L3)	3.352	2.890	0.428	4.32	706.8
Fe(L2)	0.799	3.371	0.099		718.8

$C(FeF_2)_{0.5}$_300					
	Area	FWHM	Height	Ratio	Peak Position
Fe(L3)	3.385	2.985	0.432	4.45	706.5
Fe(L2)	0.854	3.495	0.097		718.7

References

1. Cosandey, F.; Al-Sharab, J. F.; Badway, F.; Amatucci, G. G.; Stadelmann, P. *Microscopy and Microanalysis*, **2007,** *13,* 87–95.
